# Supplementary material for: A Window into the Workings of anti-B18H22 Luminescence—Blue-Fluorescent Isomeric Pair 3,3′-Cl2-B18H20 and 3,4′-Cl2-B18H20 (and Others)
Source: Molecules. 2023 Jun 1;28(11):4505. doi: 10.3390/molecules28114505 (PMC10254427; doi:10.3390/molecules28114505)
Supplement: Supplementary file 1 [file molecules-28-04505-s001.zip › molecules-2405226-supplementary.pdf]

Supporting Information

A Window into the Workings of *Anti*-$B_{18}H_{22}$ Luminescence. Blue-Fluorescent Isomeric Pair 3,3'-Cl_2-$B_{18}H_{20}$ and 3,4'-Cl_2-$B_{18}H_{20}$ (and Others)

Marcel Ehn,[a] Dmytro Bavol,[a] Jonathan Bould,[a] Vojtěch Strnad,[a,b] Miroslava Litecká,[a] Kamil Lang,[a] Kaplan Kirakci,[a] William Clegg,[c] Paul G. Waddell,[c] and Michael G. S. Londesborough.[a,*]

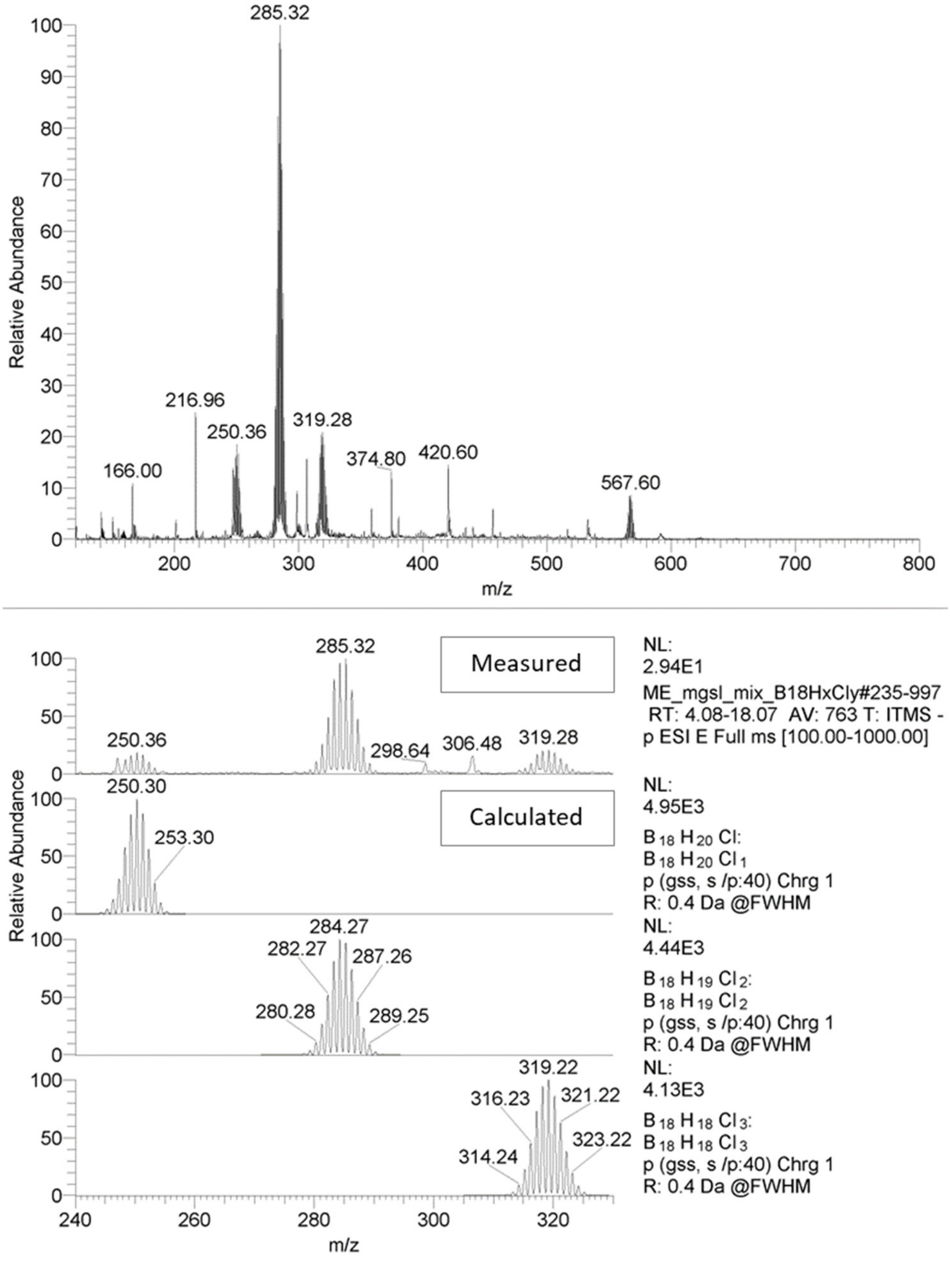

Figure S1. Mass spectrum for the reaction mixture after 5 days stirring at room temperature.

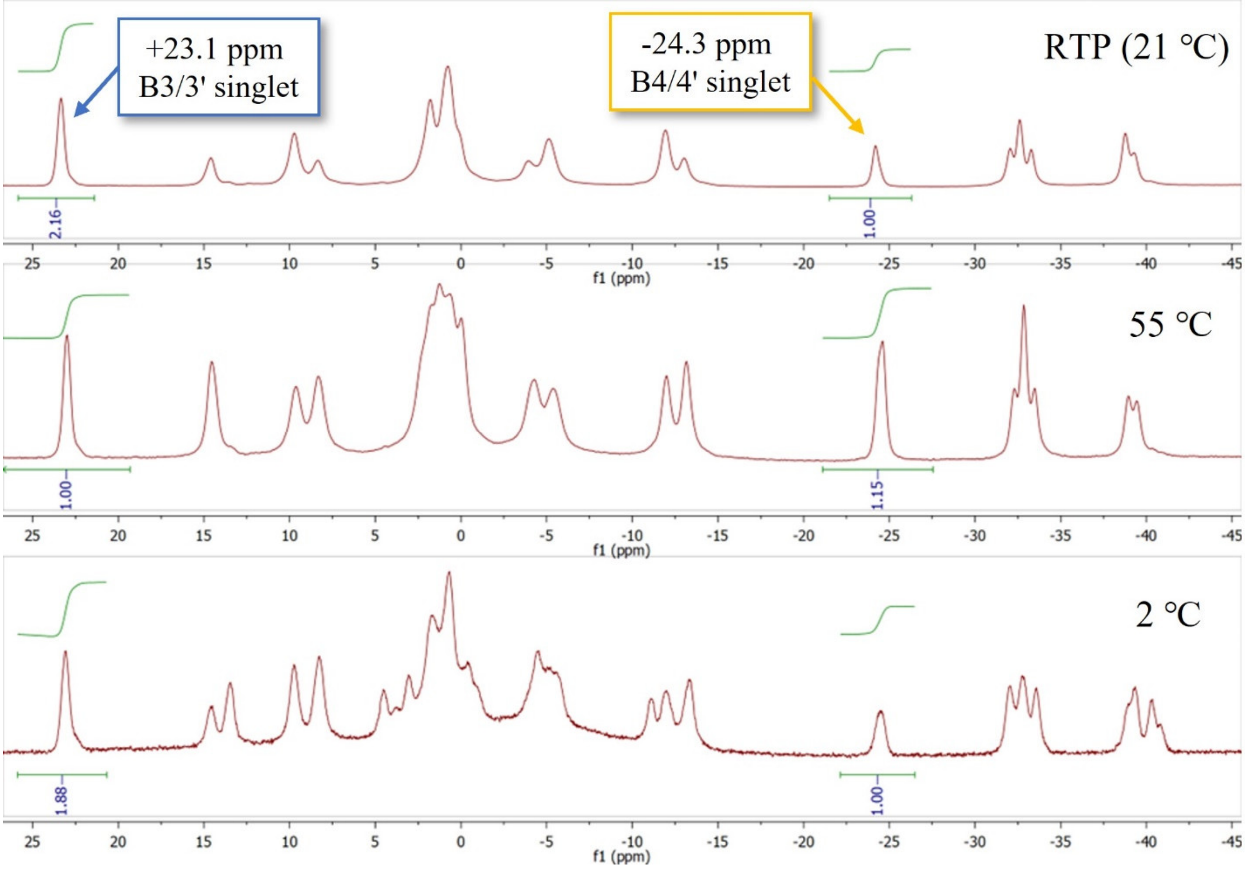

Figure S2. ^{11}B-{^{1}H} NMR spectra for the reaction mixture after (i) 5 days stirring at room temperature (21 °C), (ii) 90 mins at 55 °C, and (iii) 30 days at 2 °C. The spectra show the effect of temperature on the relative propensity for chlorination of **1** at the B3/3' and/or B4/4' positions, and also how low-temperature reaction results in significant amounts of 3-Cl-$B_{18}H_{21}$ (compound **7**) whilst RTP and higher temperature reactions form primarily 3,3'-Cl_2-$B_{18}H_{20}$ (compound **2**) and 3,4'-Cl_2-$B_{18}H_{20}$ (compound **3**).

Table S1. Interatomic distances (Å) for 3,3'-Cl_2-$B_{18}H_{20}$ (compound **2**).

B1–H1	1.038(16)	B1–B2	1.8030(18)
B1–B3	1.7742(18)	B1–B4	1.8246(19)
B1–B5	1.7520(18)	B1–B10	1.7737(19)
B2–H2	1.072(15)	B2–B3	1.7888(18)
B2–B5	1.8133(18)	B2–B5a	1.7364(18)
B2–B7	1.7798(18)	B3–Cl3	1.7918(13)
B3–B4	1.8009(19)	B3–B7	1.7435(18)
B3–B8	1.7599(19)	B4–H4	1.125(16)
B4–B8	1.7776(19)	B4–B9	1.7082(19)
B4–B10	1.7785(19)	B5–B5a	1.784(3)
B5–B7a	1.8195(18)	B5–B10	2.0279(19)

B5–H57'	1.308(17)	B7–H7	1.047(16)
B7–B8	2.0017(19)	B8–H8	1.073(16)
B8–B9	1.803(2)	B8–H89	1.256(16)
B9–H9	1.089(17)	B9–B10	1.779(2)
B9–H89	1.251(16)	B9–H910	1.273(17)
B10–H10	1.053(16)	B10–H910	1.256(17)
B11–H11	1.047(15)	B11–B12	1.7877(18)
B11–B13	1.8142(18)	B11–B14	1.8135(19)
B11–B15	1.7554(18)	B11–B20	1.7538(19)
B12–H12	1.060(15)	B12–B13	1.7815(18)
B12–B15	1.8317(19)	B12–B15b	1.7422(18)
B12–B17	1.7814(18)	B13–Cl13	1.8211(13)
B13–B14	1.7846(19)	B13–B17	1.7367(18)
B13–B18	1.7418(19)	B14–H14	1.058(17)
B14–B18	1.7867(19)	B14–B19	1.706(2)
B14–B20	1.813(2)	B15–B15b	1.800(3)
B15–B17b	1.8439(19)	B15–B20	1.9785(19)
B15–H157	1.302(17)	B17–H17	1.051(16)
B17–B18	1.969(2)	B18–H18	1.073(17)
B18–B19	1.824(2)	B18–H189	1.260(19)
B19–H19	1.043(17)	B19–B20	1.783(2)
B19–H189	1.269(18)	B19–H192	1.284(18)
B20–H20	1.068(16)	B20–H192	1.227(18)

Symmetry operations for equivalent atoms
a –x,–y+1,–z b –x+1,–y+1,–z+1

Table S2. Interatomic distances (Å) for 3,4'-Cl_2-$B_{18}H_{20}$ (compound **3**).

B1–H1	1.03(4)	B1–B2	1.803(5)
B1–B3	1.788(5)	B1–B4	1.804(5)
B1–B5	1.746(5)	B1–B10	1.749(5)
B2–H2	1.01(4)	B2–B3	1.768(5)
B2–B5	1.797(4)	B2–B6	1.754(5)
B2–B7	1.804(5)	B3–Cl3	1.805(4)
B3–B4	1.785(5)	B3–B7	1.760(6)
B3–B8	1.767(5)	B4–H4	1.182(12)
B4–B8	1.797(6)	B4–B9	1.724(7)
B4–B10	1.770(6)	B5–B6	1.805(5)
B5–B10	1.978(4)	B5–B2'	1.774(5)
B5–B7'	1.810(5)	B5–H57'	1.29(3)
B6–B7	1.817(5)	B6–H67	1.43(4)

B6–B1'	1.756(5)	B6–B2'	1.803(4)
B6–B10'	1.987(4)	B7–H7	1.13(4)
B7–B8	1.964(5)	B7–H67	1.27(4)
B8–H8	1.14(5)	B8–B9	1.783(6)
B8–H89	1.16(5)	B9–H9	1.07(4)
B9–B10	1.776(6)	B9–H89	1.22(4)
B9–H910	1.16(4)	B10–H10	1.18(4)
B10–H910	1.24(5)	B1'–H1'	1.08(4)
B1'–B2'	1.775(5)	B1'–B3'	1.790(5)
B1'–B4'	1.797(5)	B1'–B10'	1.755(5)
B2'–H2'	1.13(4)	B2'–B3'	1.747(4)
B2'–B7'	1.800(5)	B3'–H3'	1.18(4)
B3'–B4'	1.774(5)	B3'–B7'	1.752(5)
B3'–B8'	1.752(4)	B4'–Cl4'	1.804(4)
B4'–B8'	1.795(5)	B4'–B9'	1.715(5)
B4'–B10'	1.786(5)	B7'–H7'	1.17(4)
B7'–B8'	1.945(5)	B7'–H57'	1.22(4)
B8'–H8'	1.08(4)	B8'–B9'	1.787(5)
B8'–H89'	1.17(4)	B9'–H9'	1.02(4)
B9'–B10'	1.786(6)	B9'–H89'	1.21(4)
B9'–H91'	1.29(3)	B10'–H10'	1.06(5)
B10'–H91'	1.24(4)		

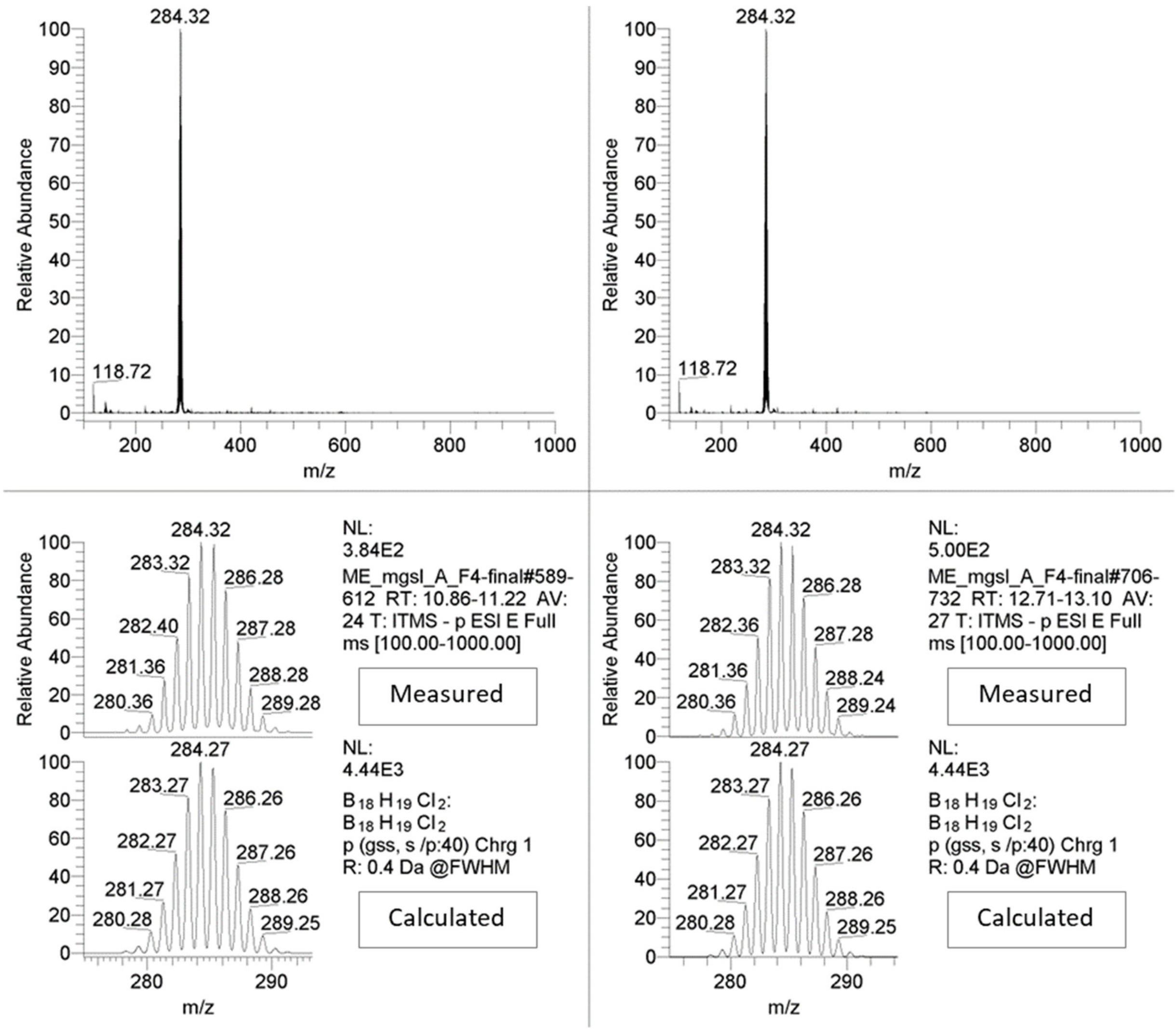

Figure S3. Mass spectra of 3,3'-Cl_2-$B_{18}H_{20}$ - left (compound **2**) and 3,4'-Cl_2-$B_{18}H_{20}$ - right (compound **3**).

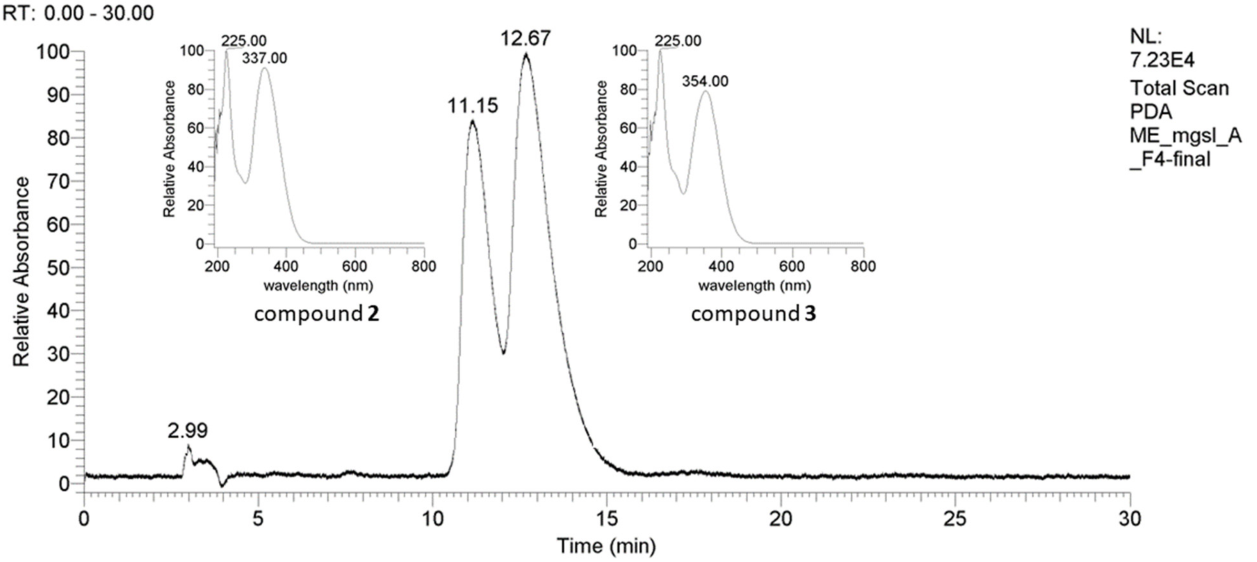

Figure S4. HPLC chromatogram of separation of 3,3'-Cl_2-$B_{18}H_{20}$; t_R = 11.15 min (compound **2**) and

3,4'-Cl_2-$B_{18}H_{20}$; t_R = 12.67 min (compound **3**); and their absorption spectra.

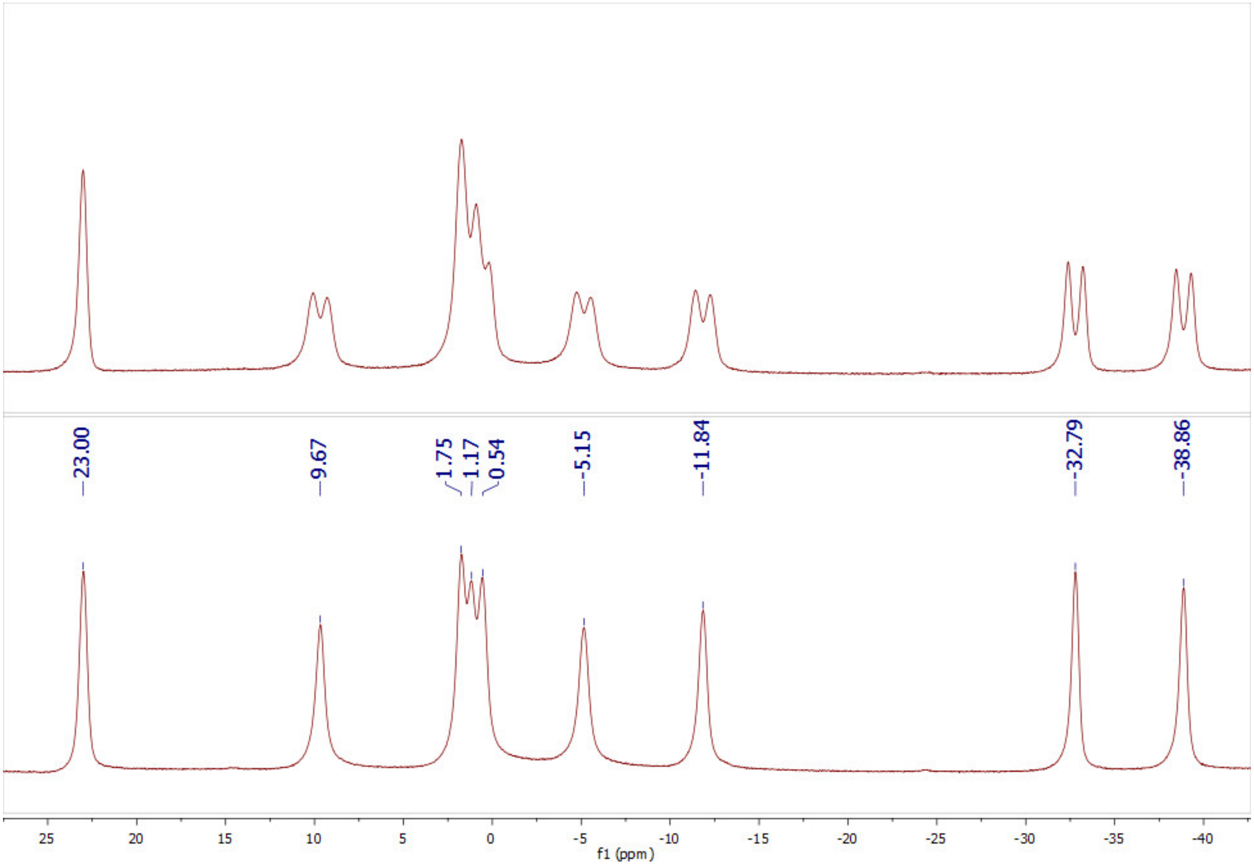

Figure S5. ^{11}B and ^{11}B-{^1H} NMR spectra for 3,3'-Cl_2-$B_{18}H_{20}$ (compound **2**).

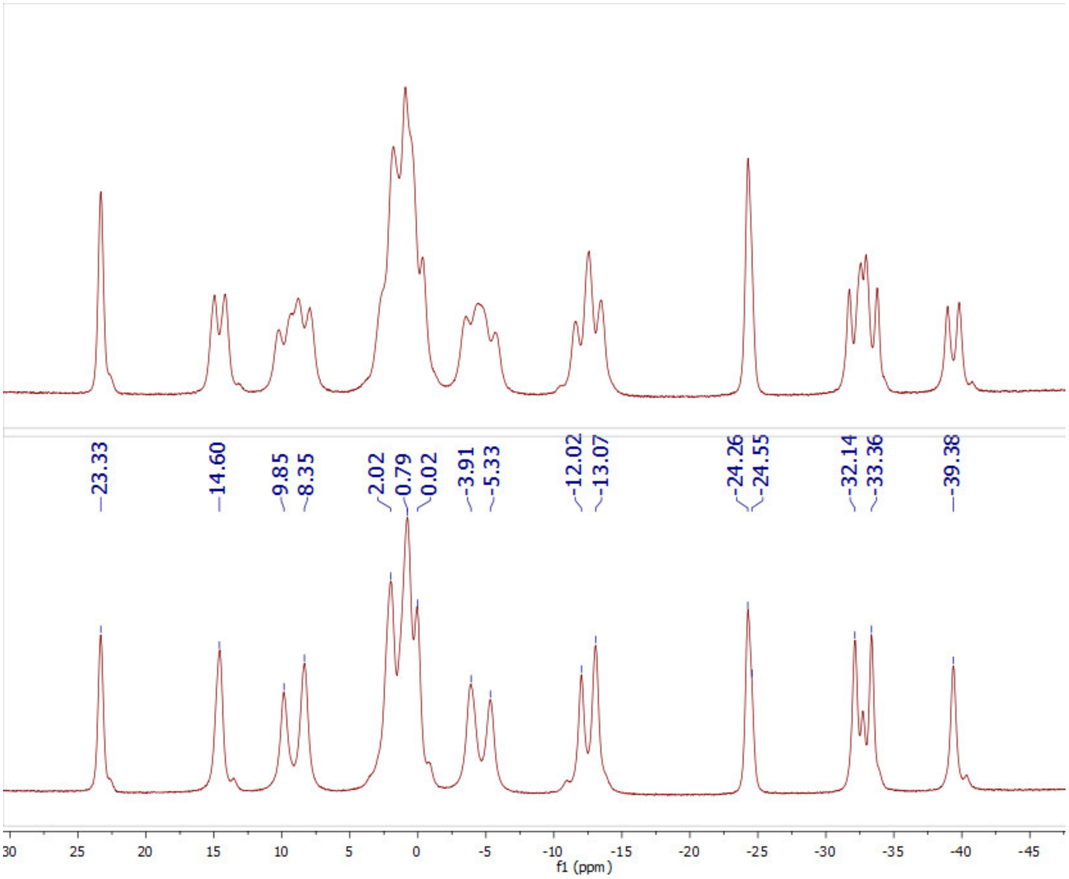

Figure S6. ^{11}B and ^{11}B-{^{1}H} NMR spectra for 3,4'-Cl_2-$B_{18}H_{20}$ (compound **3**). These spectra contain 3,3'-Cl_2-$B_{18}H_{20}$ (compound **2**) impurities.

Table S3. Interatomic distances (Å) for 4,4'-Cl_2-$B_{18}H_{20}$ (compound **4**).

B1–H1	1.100(10)	B1–B2	1.783(18)
B1–B3	1.823(17)	B1–B4	1.789(16)
B1–B5	1.761(16)	B1–B10	1.748(17)
B2–H2	1.100(10)	B2–B3	1.736(16)
B2–B5	1.833(19)	B2–B5a	1.786(17)
B2–B7	1.806(17)	B3–H3	1.099(10)
B3–B4	1.795(18)	B3–B7	1.755(17)
B3–B8	1.777(16)	B4–Cl4	1.808(11)
B4–B8	1.801(17)	B4–B9	1.719(17)
B4–B10	1.778(17)	B5–B5a	1.85(3)
B5–B7a	1.806(17)	B5–B10	1.979(17)
B5–H57	1.32(4)	B7–H7	1.100(10)
B7–B8	1.989(18)	B8–H8	1.099(10)
B8–B9	1.789(19)	B8–H89	1.32(4)
B9–H9	1.100(10)	B9–B10	1.771(16)
B9–H89	1.32(4)	B9–H91	1.32(4)

B10–H10	1.100(10)	B10–H91	1.32(4)
B11–H11	1.100(10)	B11–B12	1.806(18)
B11–B13	1.782(19)	B11–B14	1.785(17)
B11–B15	1.792(16)	B11–B20	1.766(18)
B12–H12	1.099(10)	B12–B13	1.768(16)
B12–B15	1.813(18)	B12–B15b	1.748(16)
B12–B17	1.784(17)	B13–H13	1.099(10)
B13–B14	1.776(18)	B13–B17	1.755(17)
B13–B18	1.755(17)	B14–Cl14	1.800(12)
B14–B18	1.821(19)	B14–B19	1.722(17)
B14–B20	1.774(18)	B15–B15b	1.83(2)
B15–B17b	1.798(16)	B15–B20	1.991(17)
B15–H157	1.32(4)	B17–H17	1.099(10)
B17–B18	1.974(17)	B18–H18	1.100(10)
B18–B19	1.802(19)	B18–H189	1.32(4)
B19–H19	1.100(10)	B19–B20	1.783(18)
B19–H189	1.32(4)	B19–H191	1.32(4)
B20–H20	1.100(10)	B20–H191	1.32(4)
B21–H21	1.100(10)	B21–B22	1.805(19)
B21–B23	1.826(18)	B21–B24	1.782(17)
B21–B25	1.737(17)	B21–B30	1.737(18)
B22–H22	1.101(10)	B22–B23	1.762(18)
B22–B25	1.803(19)	B22–B25c	1.789(17)
B22–B27	1.819(17)	B23–H23	1.100(10)
B23–B24	1.77(2)	B23–B27	1.783(18)
B23–B28	1.748(17)	B24–Cl24	1.820(13)
B24–B28	1.803(18)	B24–B29	1.738(18)
B24–B30	1.783(19)	B25–B25c	1.80(3)
B25–B27c	1.830(18)	B25–B30	1.992(19)
B25–H257	1.32(4)	B27–H27	1.100(10)
B27–B28	1.961(19)	B28–H28	1.100(10)
B28–B29	1.80(2)	B28–H289	1.32(4)
B29–H29	1.100(10)	B29–B30	1.806(17)
B29–H289	1.32(4)	B29–H291	1.32(4)
B30–H30	1.100(10)	B30–H291	1.32(4)
B31–H31	1.099(10)	B31–B32	1.797(18)
B31–B33	1.790(18)	B31–B34	1.800(17)
B31–B35	1.776(16)	B31–B40	1.763(18)
B32–H32	1.099(10)	B32–B33	1.769(16)
B32–B35	1.808(18)	B32–B35d	1.766(16)
B32–B37	1.805(17)	B33–H33	1.099(10)
B33–B34	1.78(2)	B33–B37	1.765(17)
B33–B38	1.734(17)	B34–Cl34	1.788(12)

B34–B38	1.78(2)	B34–B39	1.699(17)
B34–B40	1.783(19)	B35–B35d	1.79(2)
B35–B37d	1.819(17)	B35–B40	1.994(17)
B35–H357	1.32(4)	B37–H37	1.100(11)
B37–B38	1.966(19)	B38–H38	1.100(10)
B38–B39	1.78(2)	B38–H389	1.32(4)
B39–H39	1.100(10)	B39–B40	1.772(18)
B39–H389	1.32(4)	B39–H391	1.32(4)
B40–H40	1.101(10)	B40–H391	1.32(4)

Symmetry operations for equivalent atoms
a –x+2,–y+2,–z+1 b –x+1,–y,–z c –x+1,–y+1,–z+1 d –x+2,–y+1,–z+2

Table S4. Interatomic distances (Å) for 3,1'-Cl_2-$B_{18}H_{20}$ (compound **5**).

B1–H1	1.120	B1–Cl1	1.619(6)
B1–B2	1.805(8)	B1–B3	1.775(9)
B1–B4	1.787(10)	B1–B5	1.759(7)
B1–B10	1.744(10)	B2–H2	1.098(15)
B2–B3	1.772(8)	B2–B5	1.793(8)
B2–B6	1.745(7)	B2–B7	1.787(8)
B3–H3	1.120	B3–Cl3	1.795(6)
B3–B4	1.786(10)	B3–B7	1.747(8)
B3–B8	1.746(10)	B4–H4	1.098(14)
B4–B8	1.818(10)	B4–B9	1.724(12)
B4–B10	1.759(10)	B5–B6	1.804(6)
B5–B10	1.982(10)	B5–B2'	1.776(7)
B5–B7'	1.813(9)	B5–H57'	1.236(17)
B6–B7	1.810(8)	B6–B1'	1.760(7)
B6–B2'	1.802(8)	B6–B10'	2.021(9)
B6–H67	1.234(18)	B7–H7	1.095(14)
B7–B8	1.944(10)	B7–H67	1.233(18)
B8–H8	1.100(15)	B8–B9	1.802(12)
B8–H89	1.231(17)	B9–H9	1.095(14)
B9–B10	1.792(11)	B9–H89	1.233(18)
B9–H910	1.232(18)	B10–H10	1.098(15)
B10–H910	1.237(18)	B1'–H1'	1.120
B1'–Cl1'	1.682(6)	B1'–B2'	1.772(9)
B1'–B3'	1.779(9)	B1'–B4'	1.782(9)
B1'–B10'	1.767(10)	B2'–H2'	1.097(14)
B2'–B3'	1.752(9)	B2'–B7'	1.783(9)
B3'–H3'	1.120	B3'–Cl3'	1.778(7)
B3'–B4'	1.742(12)	B3'–B7'	1.770(9)

B3'–B8'	1.748(11)	B4'–H4'	1.095(14)
B4'–B8'	1.754(12)	B4'–B9'	1.723(11)
B4'–B10'	1.790(10)	B7'–H7'	1.096(14)
B7'–B8'	2.012(10)	B7'–H57'	1.234(17)
B8'–H8'	1.096(14)	B8'–B9'	1.766(12)
B8'–H89'	1.234(18)	B9'–H9'	1.096(14)
B9'–B10'	1.749(11)	B9'–H89'	1.234(18)
B9'–H91'	1.231(18)	B10'–H10'	1.108(14)
B10'–H91'	1.231(18)	Cl4–H4	0.98(6)
Cl4'–H4'	0.87(5)		

Table S5. Interatomic distances (Å) for 7,3'-Cl_2-$B_{18}H_{20}$ (compound **6**).

B1–H1	1.04(2)	B1–B2	1.790(2)
B1–B3	1.789(2)	B1–B4	1.799(2)
B1–B5	1.760(2)	B1–B10	1.751(2)
B2–H2	1.069(18)	B2–B3	1.771(2)
B2–B5	1.818(2)	B2–B6	1.762(2)
B2–B7	1.803(2)	B3–H3	1.143(18)
B3–B4	1.780(2)	B3–B7	1.760(2)
B3–B8	1.761(2)	B4–H4	1.23(2)
B4–B8	1.796(2)	B4–B9	1.725(2)
B4–B10	1.791(2)	B5–B6	1.805(2)
B5–B10	1.967(2)	B5–B2'	1.771(2)
B5–B7'	1.822(2)	B5–H57'	1.28(2)
B6–B7	1.821(2)	B6–B1'	1.759(2)
B6–B2'	1.795(2)	B6–B10'	1.996(2)
B6–H67	1.31(2)	B7–Cl7	1.7952(16)
B7–B8	1.968(2)	B7–H67	1.24(2)
B8–H8	1.09(2)	B8–B9	1.792(2)
B8–H89	1.230(19)	B9–H9	1.05(2)
B9–B10	1.781(2)	B9–H89	1.25(2)
B9–H91	1.24(2)	B10–H10	1.06(2)
B10–H91	1.26(2)	B1'–H1'	1.09(2)
B1'–B2'	1.802(2)	B1'–B3'	1.780(2)
B1'–B4'	1.802(2)	B1'–B10'	1.757(2)
B2'–H2'	1.06(2)	B2'–B3'	1.762(2)
B2'–B7'	1.793(2)	B3'–Cl3'	1.8050(17)
B3'–B4'	1.776(2)	B3'–B7'	1.771(2)
B3'–B8'	1.763(2)	B4'–H4'	1.26(2)
B4'–B8'	1.804(3)	B4'–B9'	1.722(3)
B4'–B10'	1.779(2)	B7'–H7'	1.08(2)

B7'–B8'	1.958(2)	B7'–H57'	1.26(2)
B8'–H8'	1.120(19)	B8'–B9'	1.798(2)
B8'–H89'	1.23(2)	B9'–H9'	1.09(2)
B9'–B10'	1.784(3)	B9'–H89'	1.26(2)
B9'–H91'	1.23(2)	B10'–H10'	1.05(2)
B10'–H91'	1.25(2)		

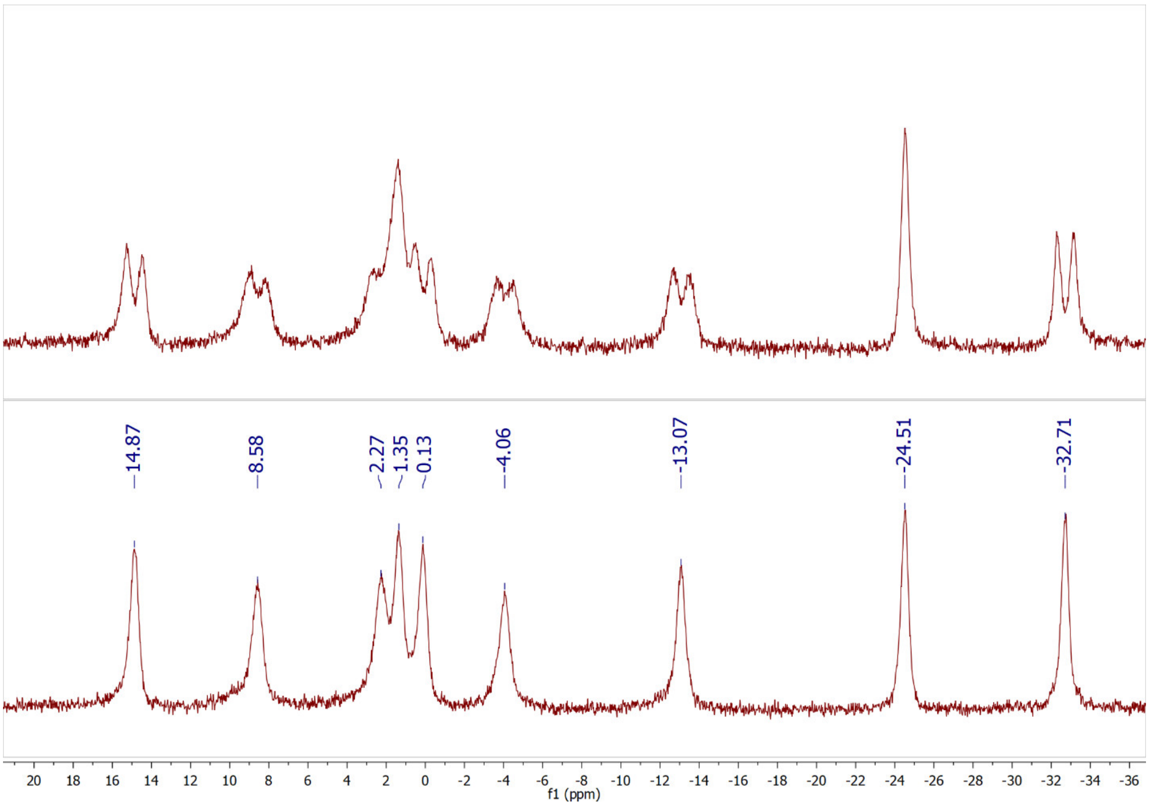

Figure S7. ^{11}B and ^{11}B-{^{1}H} NMR spectra for 4,4'-Cl_2-$B_{18}H_{20}$ (compound **4**).

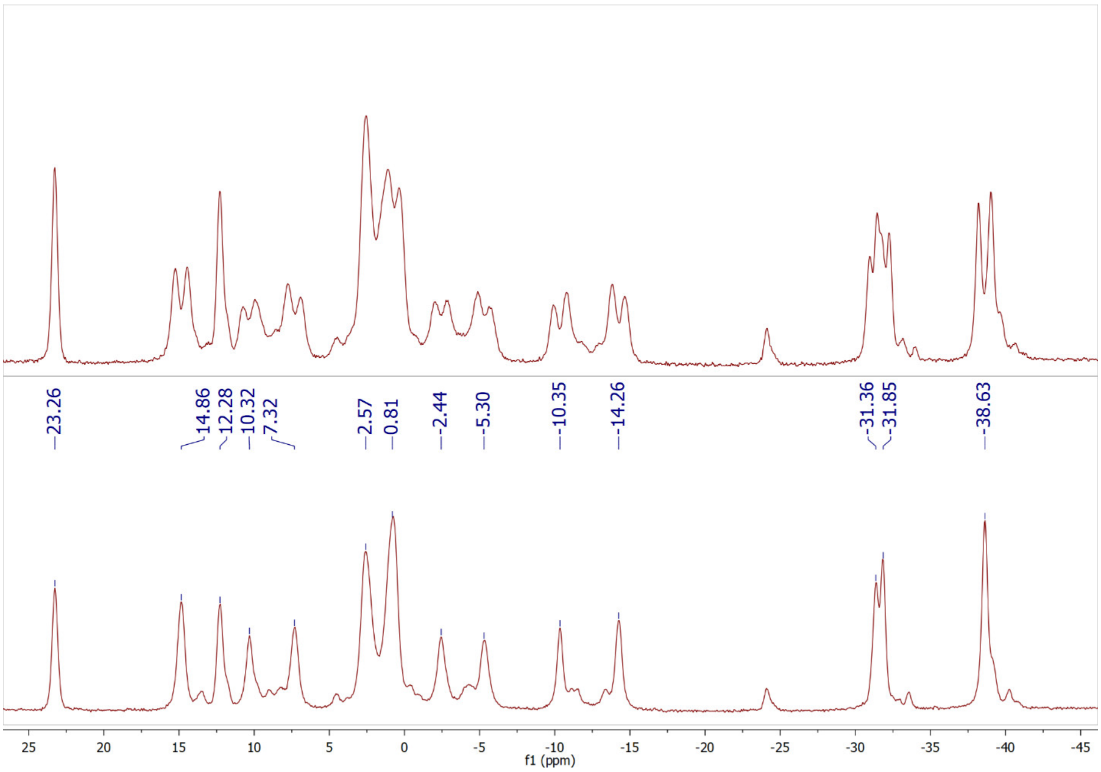

Figure S8. ^{11}B and ^{11}B-{^{1}H} NMR spectra for 3,1'-Cl_2-$B_{18}H_{20}$ (compound **5**). These spectra contain 3-Cl-$B_{18}H_{21}$ (compound **7**) impurities.

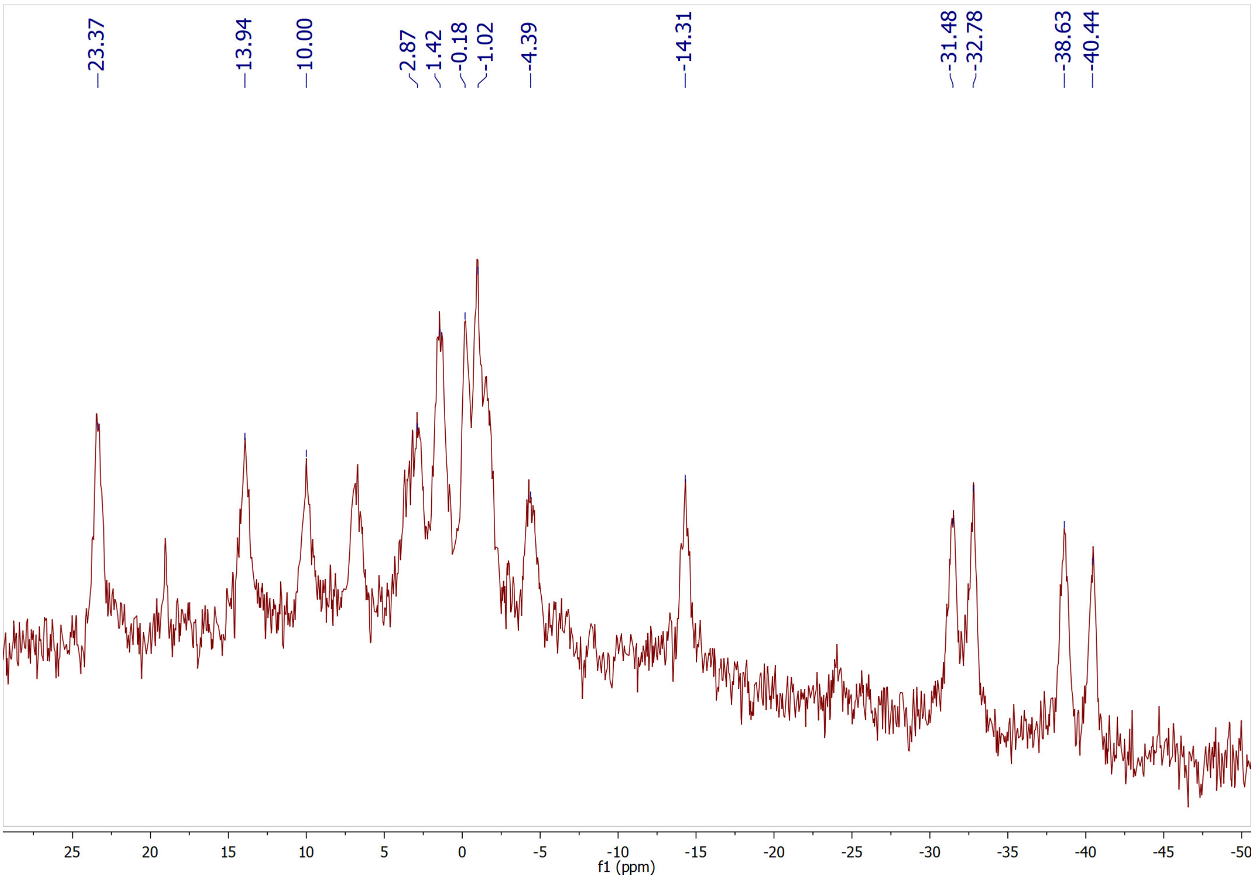

Figure S9. $^{11}B-\{^1H\}$ NMR spectrum for 7,3'-Cl_2-$B_{18}H_{20}$ (compound **6**).

Table S6. Interatomic distances (Å) for 3-Cl-$B_{18}H_{21}$ and 4-Cl-$B_{18}H_{21}$ (compounds **7** and **8**).

B1–H1	1.08(2)	B1–B2	1.781(3)
B1–B3	1.786(3)	B1–B4	1.794(3)
B1–B5a	1.754(4)	B1–B10	1.755(4)
B2–H2	1.15(2)	B2–B3	1.756(4)
B2–B5	1.764(4)	B2–B5a	1.811(3)
B2–B7	1.789(3)	B3–H3	1.120
B3–Cl3	1.730(4)	B3–B4	1.779(3)
B3–B7	1.746(4)	B3–B8	1.749(3)
B4–H4	1.120	B4–Cl4	1.681(3)
B4–B8	1.799(4)	B4–B9	1.713(4)
B4–B10	1.774(4)	B5–B5a	1.802(4)
B5–B7	1.820(3)	B5–B10a	1.972(3)
B5–H57	1.29(2)	B7–H7	1.12(2)
B7–B8	1.966(4)	B7–H57	1.24(2)
B8–H8	1.04(2)	B8–B9	1.797(3)
B8–H89	1.31(2)	B9–H9	1.11(2)
B9–B10	1.782(3)	B9–H89	1.29(2)
B9–H910	1.27(2)	B10–H10	1.09(2)
B10–H910	1.32(2)		

Symmetry operations for equivalent atoms

a –x+1,–y+1,–z+1

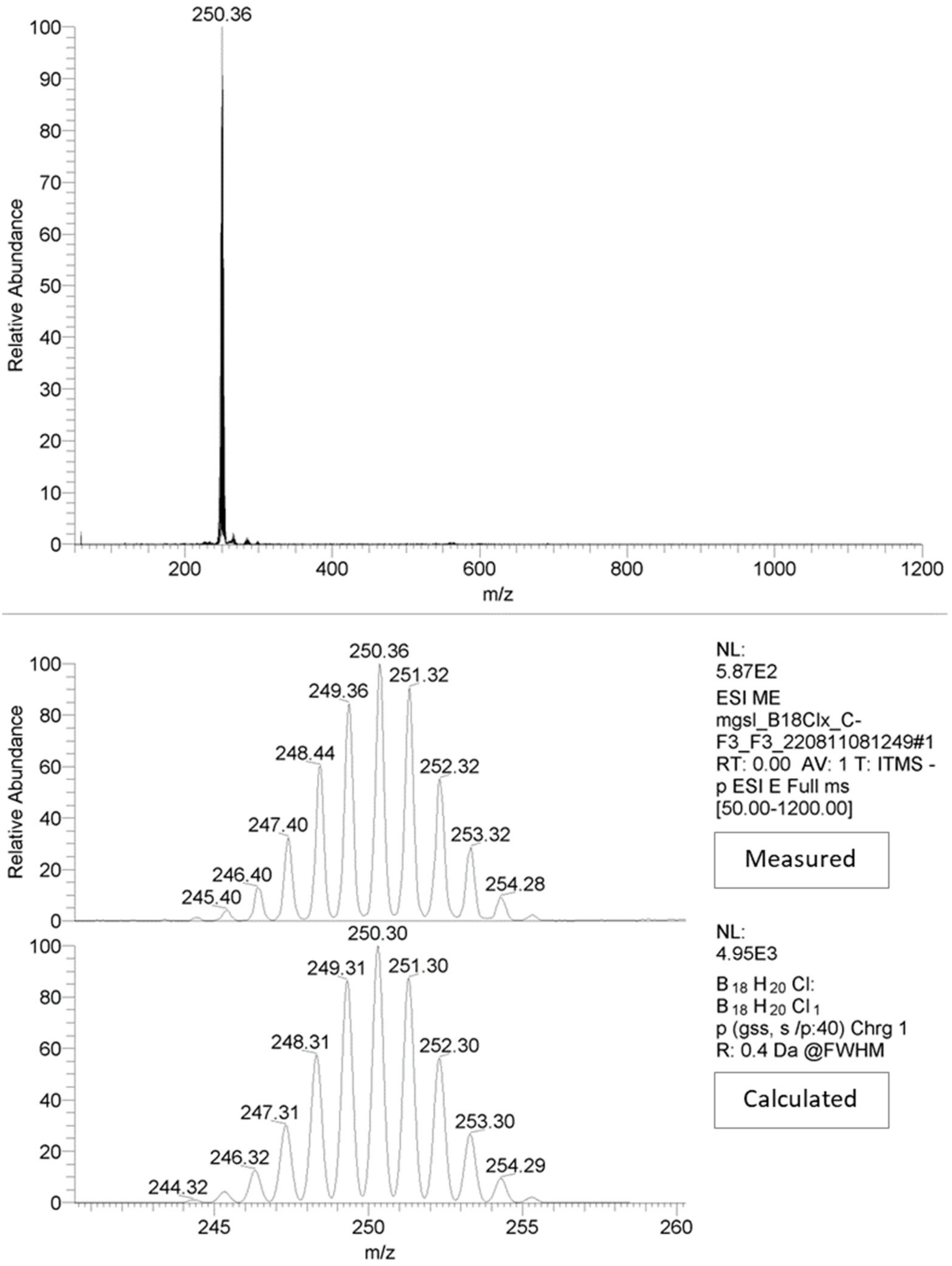

Figure S10. Mass spectrum of 3-Cl-$B_{18}H_{21}$ (compound **7** and **8**).

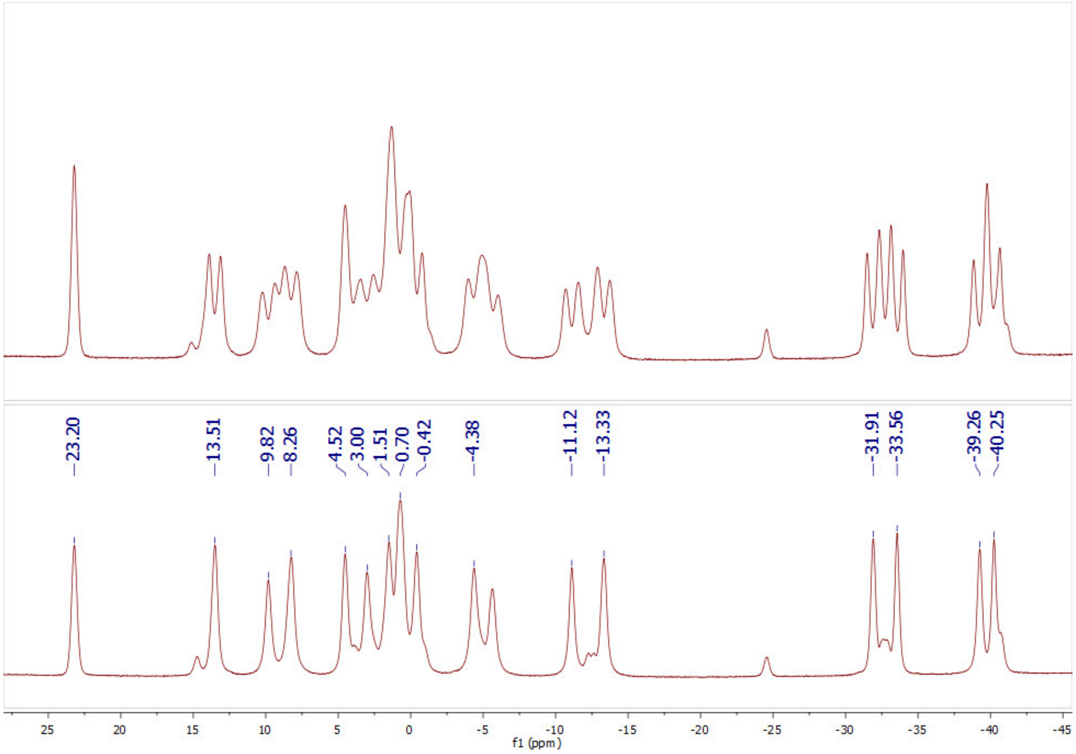

Figure S11. ^{11}B and ^{11}B-{^{1}H} NMR spectra for 3-Cl-$B_{18}H_{21}$ (compound **7**). These spectra contain 4-Cl-$B_{18}H_{21}$ (compound **8**) impurities.

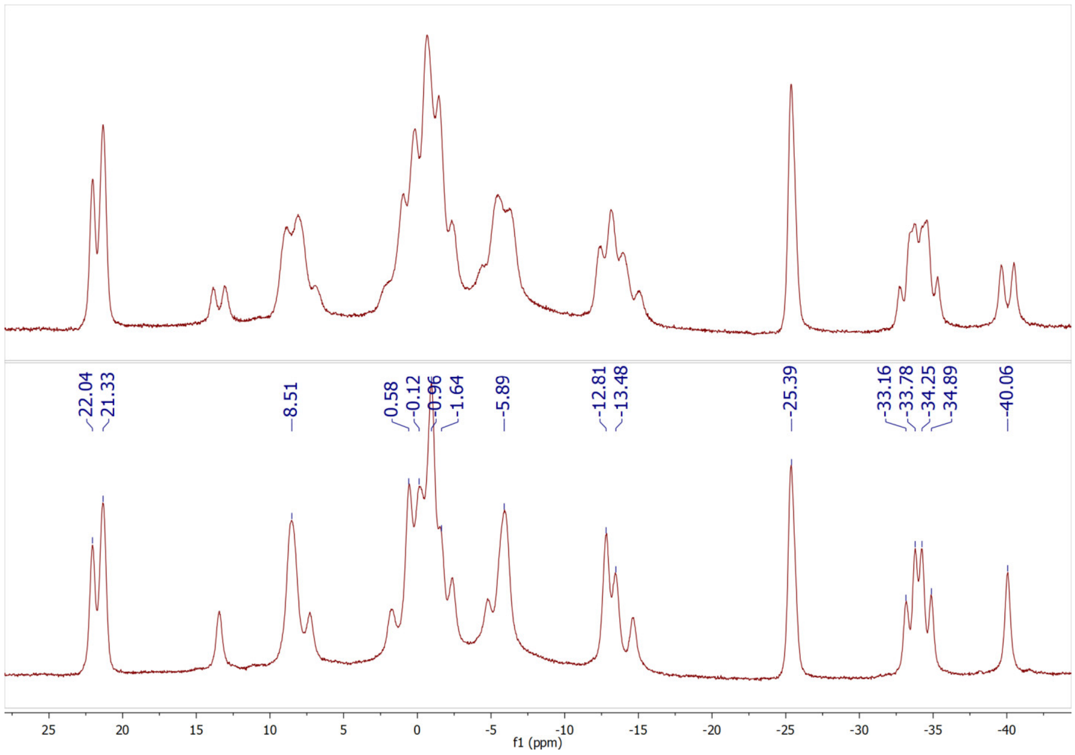

Figure S12. ^{11}B and ^{11}B-{^{1}H} NMR spectra for 3,4,3'-Cl_3-$B_{18}H_{19}$ (compound **9**). These spectra contain 3,4,4'-Cl_3-$B_{18}H_{19}$ (compound **10**) impurities.

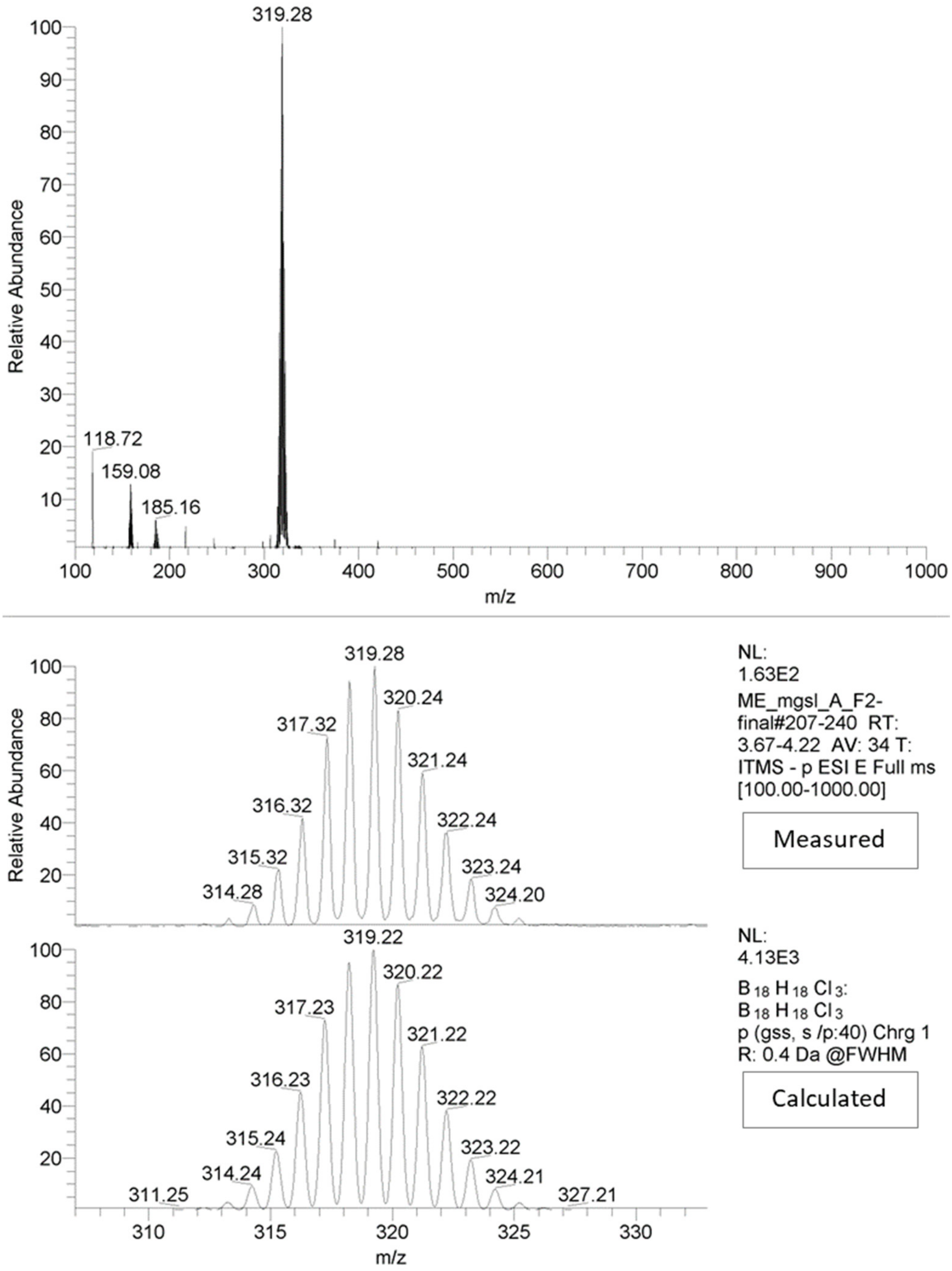

Figure S13. Mass spectrum of trichlorinated species.

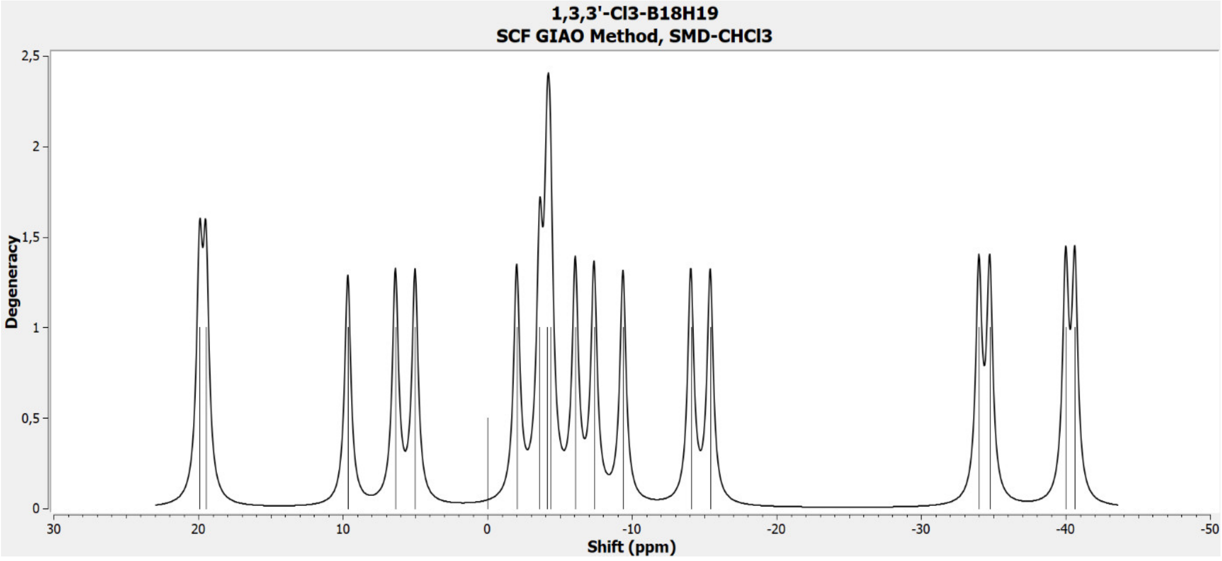

Figure S14. Calculated ^{11}B-{^{1}H} spectrum for 1,3,3'-Cl_3-$B_{18}H_{19}$.

Table S7. Calculated proton and boron-11 NMR data for 1,3,3'-Cl_3-$B_{18}H_{19}$ in $CDCl_3$ solution.

	1,3,3'-Cl_3-$B_{18}H_{19}$	
Assignment	δ(^{11}B)/ppm	δ(^{1}H)/ppm
B3′	+19.9	––a
B3	+19.5	––a
B1	+9.7	––a
B10	+6.4	+4.77
B10′	+5.0	+4.97
B6	−2.0	––b
B9	−3.6	+3.98
B5	−4.1	––b
B1′	−4.3	+4.06
B9′	−6.1	+3.90
B8′	−7.4	+3.90
B8	−9.4	+3.90
B7′	−14.1	+3.90
B7	−15.4	+3.90
B2′	−34.0	+0.58
B2	−34.7	+0.65
B4′	−40.0	+1.15
B4	−40.6	+1.05
μH8,9		−1.82
μH8′,9′		−1.73
μH9,10		−0.10
μH9′,10′		+0.40
μH6,7		+0.24
μH5,7′		+0.24

a) Chlorine substituent, b) Site of conjunction

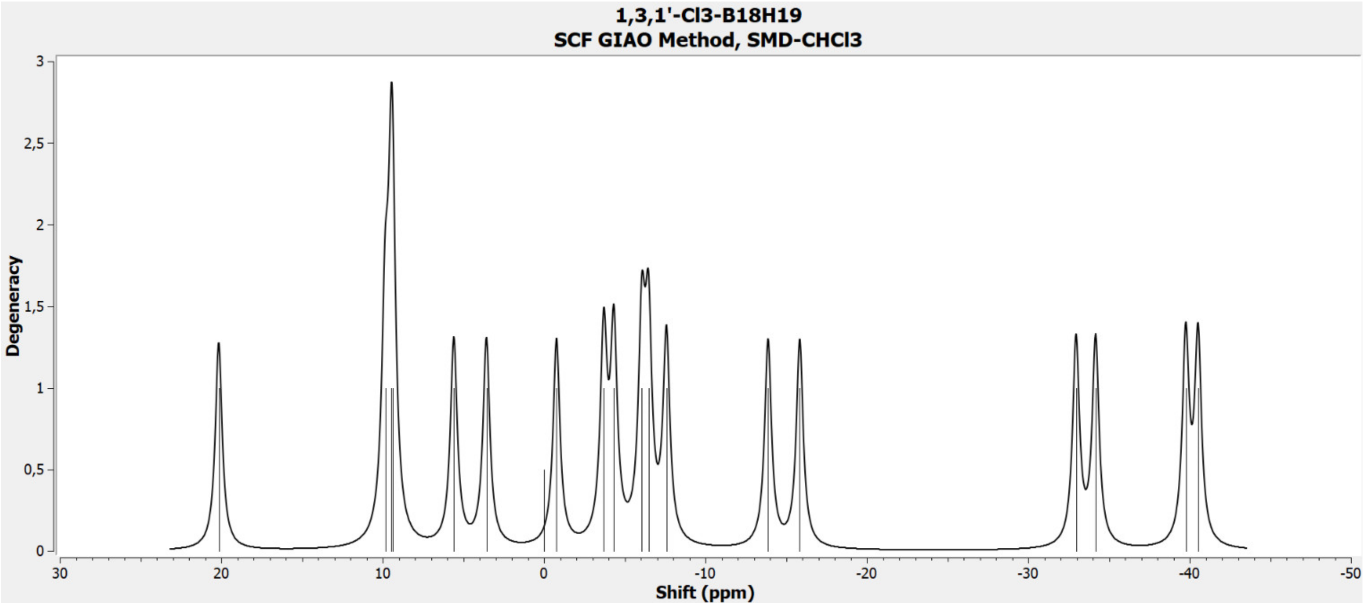

Figure S15. Calculated ^{11}B-{^{1}H} spectrum for 1,3,1'-Cl_3-$B_{18}H_{19}$.

Table S8. Calculated proton and boron-11 NMR data for 1,3,1'-Cl_3-$B_{18}H_{19}$ in $CDCl_3$ solution.

	1,3,1'-Cl_3-$B_{18}H_{19}$	
Assign-ment	δ(^{11}B)/ppm	δ(^{1}H)/ppm
B3	+20.2	----a
B1	+9.8	----a
B1′	+9.5	----a
B3′	+9.4	+4.99
B10	+5.6	+4.99
B10′	+3.6	+4.99
B5	−0.8	----b
B9′	−3.7	+3.94
B6	−4.3	----b
B9	−6.1	+3.94
B8′	−6.5	+3.79
B8	−7.6	+3.94
B7′	−13.9	+3.79
B7	−15.8	+3.94
B2	−33.0	+0.81
B2′	−34.2	+0.67
B4	−39.8	+1.20
B4′	−40.5	+1.14

μH8,9	−1.68
μH8′,9′	−2.18
μH9,10	+0.52
μH9′,10′	+0.37
μH6,7	+0.57
μH5,7′	−0.06

a) Chlorine substituent, b) Site of conjunction

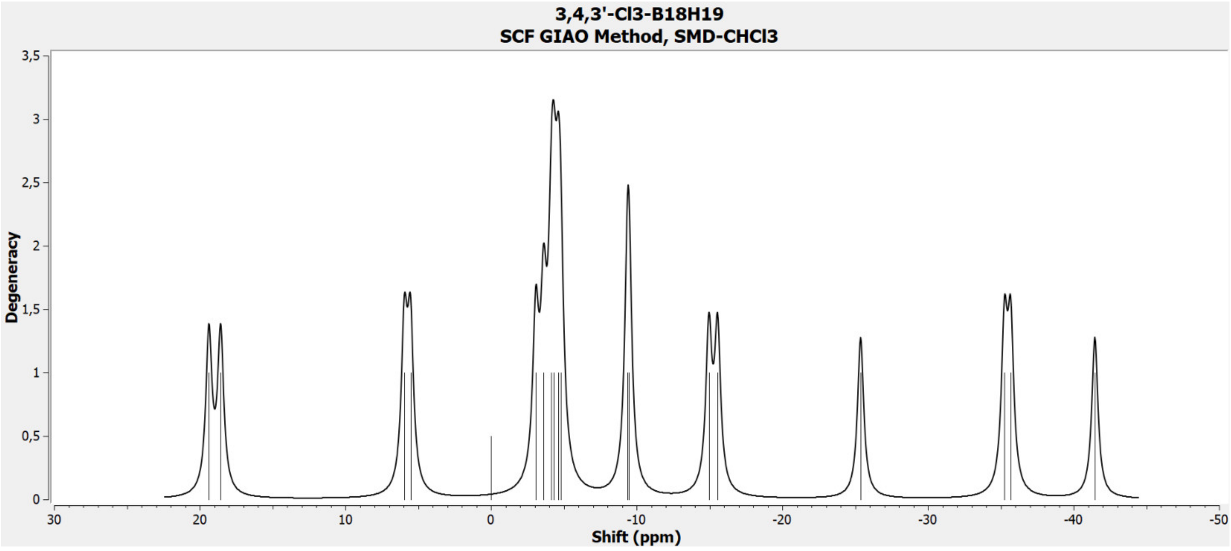

Figure S16. Calculated ^{11}B-{^{1}H} spectrum for 3,4,3'-Cl_3-$B_{18}H_{19}$ (compound **9**).

Table S9. Calculated proton and boron-11 NMR data for 3,4,3'-Cl_3-$B_{18}H_{19}$ in $CDCl_3$ solution.

	3,4,3′-Cl_3-$B_{18}H_{19}$	
Assignment	δ(^{11}B)/ppm	δ(^{1}H)/ppm
B3′	+19.4	---a
B3	+18.6	---a
B10′	+5.9	+4.74
B10	+5.5	+5.00
B1	−3.1	+4.16
B9′	−3.6	+4.00
B5	−4.1	---b
B9	−4.3	+4.22
B1′	−4.6	+4.00
B6	−4.8	---b
B8	−9.4	+4.00
B8′	−9.5	+3.85
B7′	−15.0	+3.85
B7	−15.6	+3.85
B4	−25.4	---a
B2	−35.3	+0.58
B2′	−35.7	+0.36
B4′	−41.5	+0.99
μH8,9		−1.35
μH8′,9′		−1.86
μH9,10		+0.42
μH9′,10′		−0.13
μH6,7		−0.03
μH5,7′		−0.13

a) Chlorine substituent, b) Site of conjunction

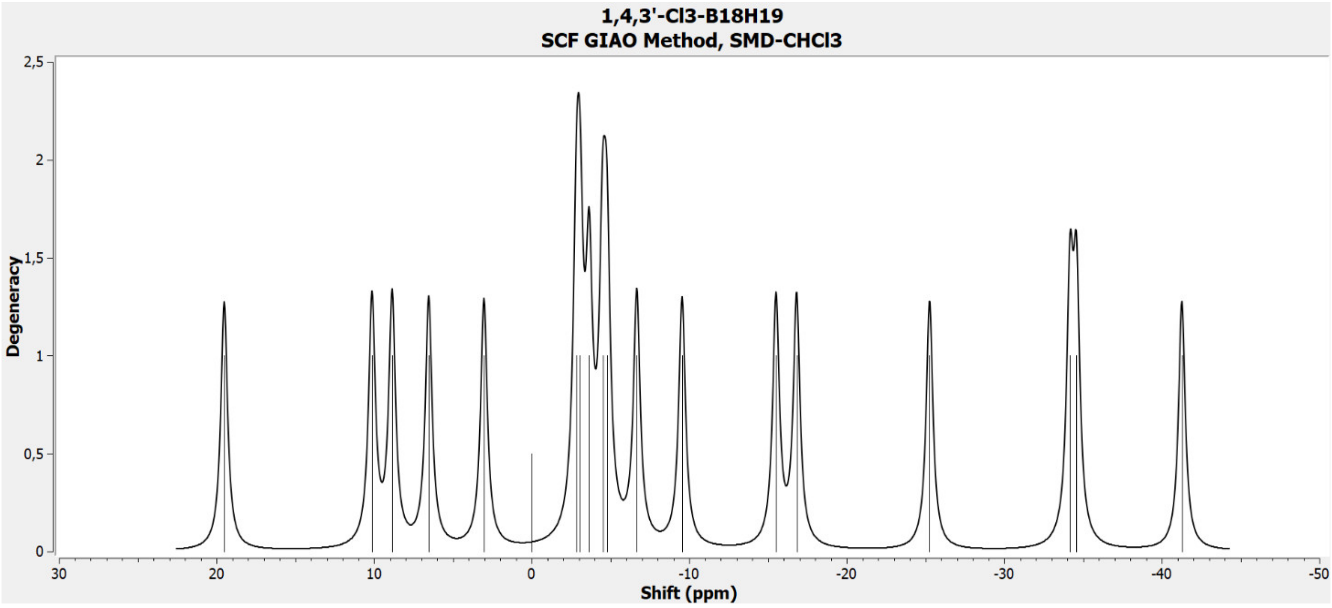

Figure S17. Calculated ^{11}B-{^{1}H} spectrum for 1,4,3'-Cl_3-$B_{18}H_{19}$.

Table S10. Calculated proton and boron-11 NMR data for 1,4,3'-Cl_3-$B_{18}H_{19}$ in $CDCl_3$ solution.

	1,4,3'-Cl_3-$B_{18}H_{19}$	
Assign-ment	δ(^{11}B)/ppm	δ(^{1}H)/ppm
B3′	+19.5	——a
B3	+10.3	+4.94
B1	+8.8	——a
B10′	+6.5	+4.78
B10	+3.0	+5.08
B5	−2.8	——b
B6	−3.1	——b
B9′	−3.7	+4.00
B9	−4.5	+4.22
B1′	−4.8	+4.00
B8	−6.7	+4.00
B8′	−9.6	+3.91
B7	−15.5	+3.63
B7′	−16.8	+3.85
B4	−25.2	——
B2′	−34.2	+0.54
B2	−34.6	+0.63
B4′	−41.3	+1.02
μH8,9		−1.60
μH8′,9′		−1.77
μH9,10		+0.76
μH9′,10′		+0.02
μH6,7		−0.40
μH5,7′		+0.43

a) Chlorine substituent, b) Site of conjunction

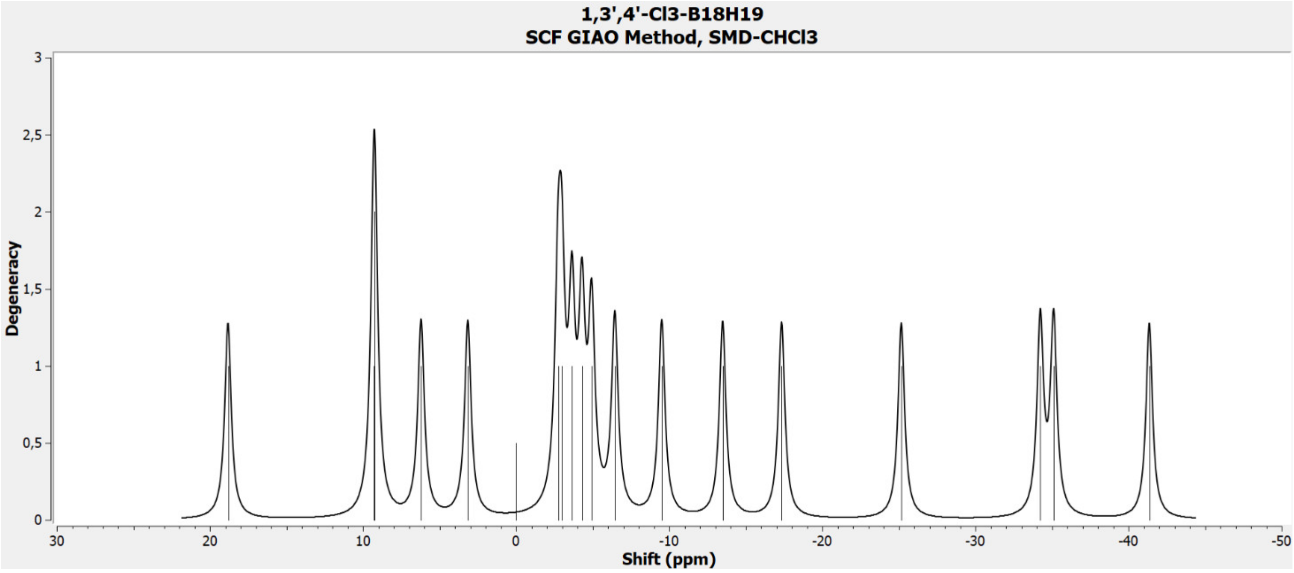

Figure S18. Calculated ^{11}B-{^{1}H} spectrum for 1,3',4'-Cl_3-$B_{18}H_{19}$.

Table S11. Calculated proton and boron-11 NMR data for 1,3',4'-Cl_3-$B_{18}H_{19}$ in $CDCl_3$ solution.

	1,3',4'-Cl_3-$B_{18}H_{19}$	
Assign-ment	δ(^{11}B)/ppm	δ(^{1}H)/ppm
B3′	+18.8	––a
B3	+9.3	+4.95
B1	+9.3	––a
B10′	+6.2	+5.05
B10	+3.2	+4.95
B6	−2.7	––b
B1′	−3.0	+4.15
B9	−3.6	+3.99
B9′	−4.3	+4.23
B5	−4.9	––b
B8	−6.4	+3.72
B8′	−9.5	+4.04
B7	−13.5	+3.72
B7′	−17.3	+3.91
B4′	−25.2	––a
B2′	−34.2	+0.86
B2	−35.1	+0.40
B4	−41.4	+1.07
μH8,9		−2.18
μH8′,9′		−1.28
μH9,10		+0.40
μH9′,10′		+0.54
μH6,7		+0.41
μH5,7′		+0.30

a) Chlorine substituent, b) Site of conjunction

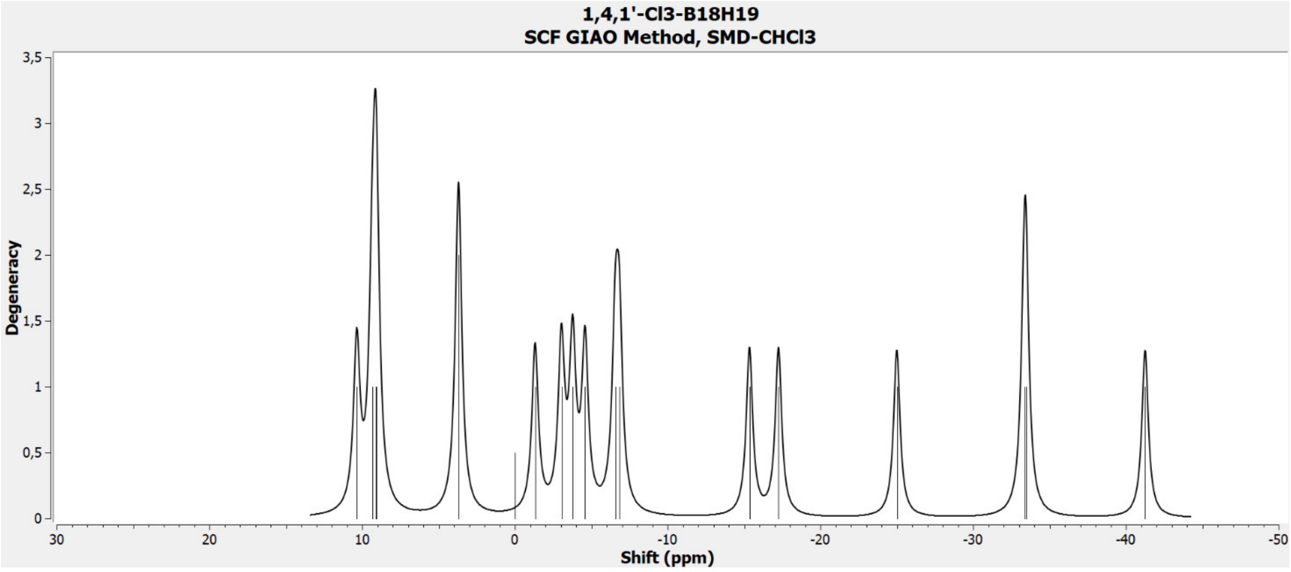

Figure S19. Calculated ^{11}B-{^{1}H} spectrum for 1,4,1'-Cl_3-$B_{18}H_{19}$.

Table S12. *Calculated proton and boron-11 NMR data for 1,4,1′-Cl_3-$B_{18}H_{19}$ in $CDCl_3$ solution.*

Assignment	1,4,1'-Cl_3-$B_{18}H_{19}$ δ(^{11}B)/ppm	δ(^{1}H)/ppm
B3	+10.4	+4.98
B3′	+9.3	+4.98
B1′	+9.1	—a
B1	+9.1	—a
B10′	+3.7	+4.98
B10	+3.7	+5.12
B5	−1.3	—b
B6	−3.0	—b
B9′	−3.8	+4.02
B9	−4.6	+4.23
B8′	−6.6	+3.75
B8	−6.8	+4.02
B7′	−15.4	+3.75
B7	−17.3	+3.67
B4	−25.0	—a
B2	−33.4	+0.87
B2′	−33.5	+0.58
B4′	−41.3	+1.10
μH8,9		−1.52
μH8′,9′		−2.09
μH9,10		+0.87
μH9′,10′		+0.49
μH6,7		-0.09
μH5,7′		+0.15

a) Chlorine substituent, b) Site of conjunction

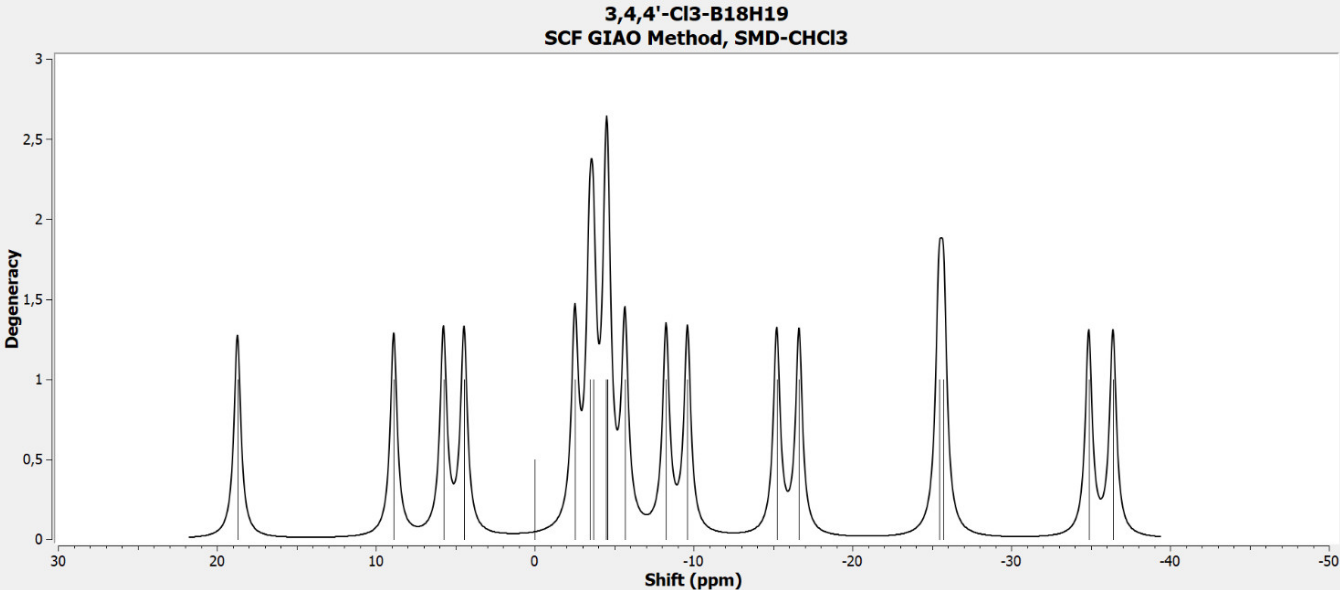

Figure S20. Calculated ^{11}B-{^{1}H} spectrum for 3,4,4'-Cl_3-$B_{18}H_{19}$ (compound **10**).

Table S13. Calculated proton and boron-11 NMR data for 3,4,4'-Cl_3-$B_{18}H_{19}$ in $CDCl_3$ solution.

Assignment	3,4,4′-Cl_3-$B_{18}H_{19}$ δ(^{11}B)/ppm	δ(^{1}H)/ppm
B3	+18.7	––a
B3′	+8.9	+4.70
B10	+5.7	+5.02
B10′	+4.5	+5.02
B9′	−2.5	+4.32
B1	−3.5	+4.02
B5	−3.7	––b
B9	−4.5	+4.23
B1′	−4.6	+3.88
B6	−5.7	––b
B8′	−8.3	+4.02
B8	−9.6	+4.02
B7′	−15.2	+3.66
B7	−16.6	+3.82
B4′	−25.4	––a
B4	−25.7	––a
B2	−34.9	+0.51
B2′	−36.4	+0.38
μH8,9		−1.30
μH8′,9′		−1.69
μH9,10		+0.51
μH9′,10′		+0.38
μH6,7		+0.03
μH5,7′		−0.67

a) Chlorine substituent, b) Site of conjunction

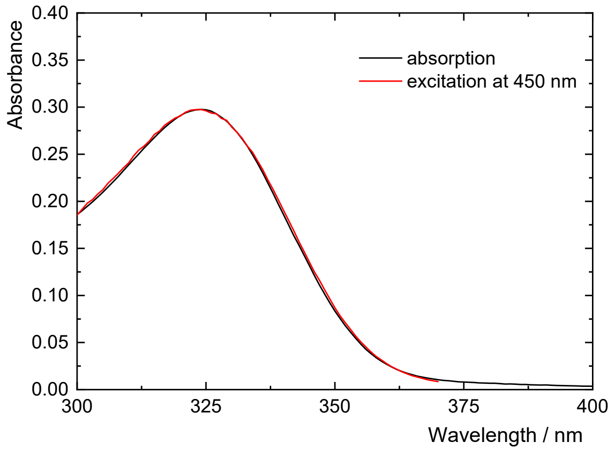

Figure S21. Absorption and excitation spectra for 3,3'-Cl_2-$B_{18}H_{20}$ (compound **2**).

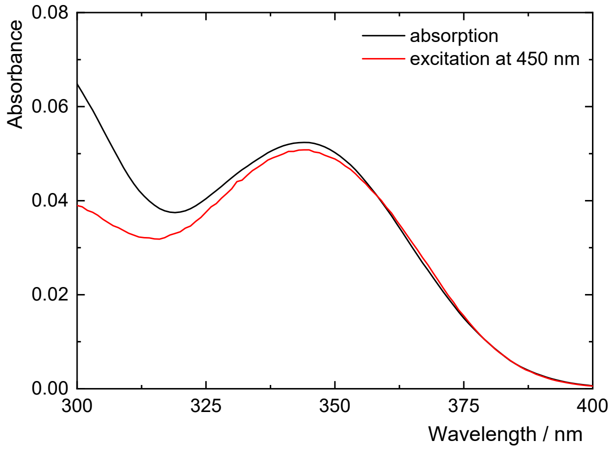

Figure S22. Absorption and excitation spectra for 4,4'-Cl_2-$B_{18}H_{20}$ (compound **4**).

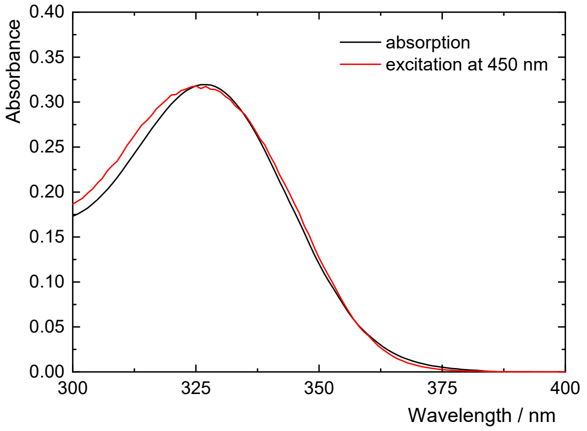

Figure S23. Absorption and excitation spectra for 3-Cl-$B_{18}H_{21}$ (compound **7**).

Table S14. Crystal and refinement data for other forms of compound **2.**

	**2a**	**2b**	**2c**
Chemical formula	$B_{18}H_{20}Cl_2$	$B_{18}H_{20}Cl_2$	$B_{18}H_{20}Cl_2$
M_r	285.6	285.6	285.6
Crystal system	triclinic	monoclinic	monoclinic
Space group	$P\bar{1}$	$P2_1/c$	$P2_1/n$
a (Å)	11.5180(2)	11.2999(3)	7.1315(5)
b (Å)	15.8456(2)	12.8306(3)	11.2652(4)
c (Å)	20.4360(2)	11.0325(3)	10.4227(6)
◎◎(°)	69.7276(8)		
◎ (°)	74.6731(14)	95.209(2)	106.413(6)
◎ (Å)	69.0113(14)		
V (Å^3)	3224.46(9)	1592.94(7)	803.21(8)
Z	8	4	2
Crystal size (mm^3)	0.21×0.11×0.07	0.15×0.10×0.01	0.09×0.07×0.05
T (K)	100	150	150
Radiation,◎◎ (Å)	CuK◎, 1.54184	CuK◎, 1.54184	CuK◎, 1.54184
Reflections measured	100755	11501	5794
Unique reflections	15567	2820	1428
R_{int}	0.1818	0.0432	0.0410
Parameters, restraints	1043, 0	267, 11	137, 9
R (F, F^2>2◎)	0.0472	0.0358	0.0374
R_w (F^2, all data)	0.1665	0.0901	0.1020
Goodness of fit (F^2)	1.067	1.026	1.037
Flack parameter			
Max, min ◎◎ (e Å^{-3})	0.64, −0.53	0.41, −0.33	0.29, −0.14
CCDC deposition number	2256516	2178829	2178830

Table S15. B-B and B-Cl bond lengths in ordered molecules of $B_{18}H_{22}$ and $B_{18}H_{20}Cl_2$. Differences from corresponding values in $B_{18}H_{22}$ are on the next page; red for Cl-substituted sites.

	B18H22	2: 3,3'	2: 3,3'	3: 3,4'	4: 4,4'	4: 4,4'	4: 4,4'	4: 4,4'	6: 7,3'
site 1	0	3	3	3	4	4	4	4	7
site 2	0	3'	3'	4'	4'	4'	4'	4'	3'
1-2	1.791	1.803	1.788	1.803	1.783	1.806	1.805	1.797	1.790
1-3	1.795	1.774	1.814	1.788	1.823	1.782	1.826	1.790	1.789
1-4	1.792	1.825	1.814	1.804	1.789	1.785	1.782	1.800	1.799
1-5	1.755	1.752	1.755	1.746	1.761	1.792	1.737	1.776	1.760
1-10	1.755	1.774	1.754	1.749	1.748	1.766	1.737	1.763	1.751
2-3	1.761	1.789	1.782	1.768	1.736	1.768	1.762	1.769	1.771
2-5	1.810	1.813	1.832	1.797	1.833	1.813	1.803	1.808	1.818
2-6	1.770	1.736	1.742	1.754	1.786	1.748	1.789	1.766	1.762
2-7	1.794	1.780	1.781	1.804	1.806	1.784	1.819	1.805	1.803
3-4	1.783	1.801	1.785	1.785	1.795	1.776	1.770	1.780	1.780
3-7	1.762	1.744	1.737	1.760	1.755	1.755	1.783	1.765	1.760
3-8	1.753	1.760	1.742	1.767	1.777	1.755	1.748	1.734	1.761
4-8	1.806	1.778	1.787	1.797	1.801	1.821	1.803	1.780	1.796
4-9	1.720	1.708	1.706	1.724	1.719	1.722	1.738	1.699	1.725
4-10	1.779	1.779	1.813	1.770	1.778	1.774	1.783	1.783	1.791
5-6	1.805	1.784	1.800	1.805	1.850	1.830	1.800	1.790	1.805
5-10	1.984	2.028	1.979	1.978	1.979	1.991	1.992	1.994	1.967
5-2'	1.770	1.736	1.742	1.774	1.786	1.748	1.789	1.766	1.771
5-7'	1.820	1.820	1.844	1.810	1.806	1.798	1.830	1.819	1.822
6-7	1.820	1.820	1.844	1.817	1.806	1.798	1.830	1.819	1.821
6-1'	1.755	1.752	1.755	1.756	1.761	1.792	1.737	1.776	1.759
6-2'	1.810	1.813	1.832	1.803	1.833	1.813	1.803	1.766	1.795
6-10'	1.984	2.028	1.979	1.987	1.979	1.991	1.992	1.994	1.996
7-8	1.970	2.002	1.969	1.964	1.989	1.974	1.961	1.966	1.968
8-9	1.802	1.803	1.824	1.783	1.789	1.802	1.800	1.780	1.792
9-10	1.783	1.779	1.783	1.776	1.771	1.783	1.806	1.772	1.781
1'-2'	1.791	1.803	1.788	1.775	1.783	1.806	1.805	1.797	1.802
1'-3'	1.795	1.774	1.814	1.790	1.823	1.782	1.826	1.790	1.780
1'-4'	1.792	1.825	1.814	1.797	1.789	1.785	1.782	1.800	1.802
1'-10'	1.755	1.774	1.754	1.755	1.748	1.766	1.737	1.763	1.757
2'-3'	1.761	1.789	1.782	1.747	1.736	1.768	1.762	1.769	1.762
2'-7'	1.794	1.780	1.781	1.800	1.806	1.784	1.819	1.805	1.793
3'-4'	1.783	1.801	1.785	1.774	1.795	1.776	1.770	1.780	1.776
3'-7'	1.762	1.744	1.737	1.752	1.755	1.755	1.783	1.765	1.771
3'-8'	1.753	1.760	1.742	1.752	1.777	1.755	1.748	1.734	1.763
4'-8'	1.806	1.778	1.787	1.795	1.801	1.821	1.803	1.780	1.804
4'-9'	1.720	1.708	1.706	1.715	1.719	1.722	1.738	1.699	1.722
4'-10'	1.779	1.779	1.813	1.786	1.778	1.774	1.783	1.783	1.779
7'-8'	1.970	2.002	1.969	1.945	1.989	1.974	1.961	1.966	1.958
8'-9'	1.802	1.803	1.824	1.787	1.789	1.802	1.800	1.780	1.798
9'-10'	1.783	1.779	1.783	1.786	1.771	1.783	1.806	1.772	1.784
B-Cl		1.792	1.821	1.805	1.808	1.800	1.820	1.788	1.795
B-Cl'		1.792	1.821	1.804	1.808	1.800	1.820	1.788	1.805

	2: 3,3'	2: 3,3'	3: 3,4'	4: 4,4'	4: 4,4'	4: 4,4'	4: 4,4'	6: 7,3'
site 1	3	3	3	4	4	4	4	7
site 2	3'	3'	4'	4'	4'	4'	4'	3'
1-2	-0.012	0.003	-0.012	0.008	-0.015	-0.014	-0.006	0.001
1-3	0.021	-0.019	0.007	-0.028	0.013	-0.031	0.005	0.006
1-4	-0.033	-0.022	-0.012	0.003	0.007	0.010	-0.008	-0.007
1-5	0.003	0.000	0.009	-0.006	-0.037	0.018	-0.021	-0.005
1-10	-0.019	0.001	0.006	0.007	-0.011	0.018	-0.008	0.004
2-3	-0.028	-0.021	-0.007	0.025	-0.007	-0.001	-0.008	-0.010
2-5	-0.003	-0.022	0.013	-0.023	-0.003	0.007	0.002	-0.008
2-6	0.034	0.028	0.016	-0.016	0.022	-0.019	0.004	0.008
2-7	0.014	0.013	-0.010	-0.012	0.010	-0.025	-0.011	-0.009
3-4	-0.018	-0.002	-0.002	-0.012	0.007	0.013	0.003	0.003
3-7	0.018	0.025	0.002	0.007	0.007	-0.021	-0.003	0.002
3-8	-0.007	0.011	-0.014	-0.024	-0.002	0.005	0.019	-0.008
4-8	0.028	0.019	0.009	0.005	-0.015	0.003	0.026	0.010
4-9	0.012	0.014	-0.004	0.001	-0.002	-0.018	0.021	-0.005
4-10	0.000	-0.034	0.009	0.001	0.005	-0.004	-0.004	-0.012
5-6	0.021	0.005	0.000	-0.045	-0.025	0.005	0.015	0.000
5-10	-0.044	0.005	0.006	0.005	-0.007	-0.008	-0.010	0.017
5-2'	0.034	0.028	-0.004	-0.016	0.022	-0.019	0.004	-0.001
5-7'	0.000	-0.024	0.010	0.014	0.022	-0.010	0.001	-0.002
6-7	0.000	-0.024	0.003	0.014	0.022	-0.010	0.001	-0.001
6-1'	0.003	0.000	-0.001	-0.006	-0.037	0.018	-0.021	-0.004
6-2'	-0.003	-0.022	0.007	-0.023	-0.003	0.007	0.044	0.015
6-10'	-0.044	0.005	-0.003	0.005	-0.007	-0.008	-0.010	-0.012
7-8	-0.032	0.001	0.006	-0.019	-0.004	0.009	0.004	0.002
8-9	-0.001	-0.022	0.019	0.013	0.000	0.002	0.022	0.010
9-10	0.004	0.000	0.007	0.012	0.000	-0.023	0.011	0.002
1'-2'	-0.012	0.003	0.016	0.008	-0.015	-0.014	-0.006	-0.011
1'-3'	0.021	-0.019	0.005	-0.028	0.013	-0.031	0.005	0.015
1'-4'	-0.033	-0.022	-0.005	0.003	0.007	0.010	-0.008	-0.010
1'-10'	-0.019	0.001	0.000	0.007	-0.011	0.018	-0.008	-0.002
2'-3'	-0.028	-0.021	0.014	0.025	-0.007	-0.001	-0.008	-0.001
2'-7'	0.014	0.013	-0.006	-0.012	0.010	-0.025	-0.011	0.001
3'-4'	-0.018	-0.002	0.009	-0.012	0.007	0.013	0.003	0.007
3'-7'	0.018	0.025	0.010	0.007	0.007	-0.021	-0.003	-0.009
3'-8'	-0.007	0.011	0.001	-0.024	-0.002	0.005	0.019	-0.010
4'-8'	0.028	0.019	0.011	0.005	-0.015	0.003	0.026	0.002
4'-9'	0.012	0.014	0.005	0.001	-0.002	-0.018	0.021	-0.002
4'-10'	0.000	-0.034	-0.007	0.001	0.005	-0.004	-0.004	0.000
7'-8'	-0.032	0.001	0.025	-0.019	-0.004	0.009	0.004	0.012
8'-9'	-0.001	-0.022	0.015	0.013	0.000	0.002	0.022	0.004
9'-10'	0.004	0.000	-0.003	0.012	0.000	-0.023	0.011	-0.001
